# How Ladies Should Ride Horseback

**Published:** 1885-08

**Authors:** 


### How Ladies should Ride Horseback.

The horsewomen should sit so that the weight of the body falls exactly in the centre of the saddle, without heavily bearing on the stirrup, able to grasp the upright pommel with the right knee, and press against the "hunting horn" with her left knee, yet not exerting any muscular action for that purpose. For this end the stirrup leather must be neither too long nor too short. The ideal of a fine horsewoman is to be erect without being rigid, square to the front, and, until quite at home in the saddle, looking religiously between her horse's ears. The shoulders must, therefore, be square, but thrown back a little so as to expand the chest and make a hollow waist, "such as is observed in waltzing," but always flexible. On flexibility of the person above the waist and on the firmness below, all the grace of equestrianism, all the safety, depsnd. Nervousness makes both men and women poke their heads forward—a stupid trick in a man, unpardonable in a woman. A lady should bend like a willow in a storm, always returning to an easy yet nearly upright position. This seat should be acquired while the lady's horse is led, first by hand, then with a leading stick and finally with a lunging rein, which will give room for cantering in circles. But where the pupil is encumbered with reins, a whip and directions for guiding her horse, she may be excused for forgetting all about her seat or her position. The arms down to the elbows should hang loosely near, but not fixed to the sides, and the hands, in the absence of reins, may rest in front of the waist.—*Philadelphia Times.*

